# Dermoscopic Follow-Up of the Skin towards Acute Graft-versus-Host-Disease in Patients after Allogeneic Hematopoietic Stem Cell Transplantation

**DOI:** 10.1155/2016/4535717

**Published:** 2016-06-30

**Authors:** Grazyna Kaminska-Winciorek, Tomasz Czerw, Tomasz Kruzel, Sebastian Giebel

**Affiliations:** Department of Bone Marrow Transplantation and Onco-Hematology, Maria Sklodowska-Curie Memorial Cancer Centre and Institute of Oncology, Gliwice Branch, 15 Wybrzeze Armii Krajowej Street, 44-101 Gliwice, Poland

## Abstract

*Background*. Acute graft-versus-host disease (aGVHD) involving skin is one of the most frequent complications of allogeneic hematopoietic stem cell transplantation (alloHSCT), usually diagnosed based on clinical manifestations. So far, skin biopsy with histopathological evaluation is the only method to confirm the diagnosis.* Objective*. In this prospective study we monitored alloHSCT recipients by dermoscopy in order to assess its utility as an alternative noninvasive tool to early diagnose acute GVHD.* Methods*. Thirteen consecutive patients who received alloHSCT were examined clinically and dermoscopically towards aGVHD [days 28 (±7), 56 (±7), and 100 (±7)], as well as in each patient who developed cutaneous aGVHD diagnosed according to clinical criteria (Glucksberg scale).* Results*. Six patients (46%) developed symptoms of cutaneous acute GVHD (grade 1, *n* = 3; grade 2, *n* = 3). Dermoscopic evaluation revealed pinkish or reddish background and well-visible, multiple thin telangiectasias.* Conclusion*. To our knowledge, this is the first report on the use of dermoscopy to evaluate skin involvement in the course of acute GVHD suggesting its role as a diagnostic tool in follow-up of GVHD, which can be also used before clinical symptoms occur.

## 1. Introduction

Acute graft-versus-host-disease (aGVHD) remains the major cause of short-term (100 days) mortality after allogeneic hematopoietic stem cell transplantation (alloHSCT) [1]. Acute GVHD is manifested by one or more of the following features: erythematous skin lesions, cholestatic liver disease, and gastrointestinal disturbances [1]. Skin manifestations vary and may include erythematous maculopapular rash, which may be pruritic or painful, in severe cases becoming bullous with subsequent desquamation [1]. The classification of aGVHD is based on Glucksberg scale in which each organ is staged from 0 to 4 [1]. The stage of skin lesions is based on the presence of maculopapular rash and the affected body surface area (<25% in stage 1, 25–50% in stage 2) with generalized erythroderma (stage 3) leading to bullae with desquamation in stage 4 [1]. The scores for all stages are combined to produce an overall grade (from I to IV) depending on organ and stage of involvement.

Using a novel aGVHD Risk Score, high risk (HR) GVHD is defined as either skin stage 4, lower gastrointestinal (GI) stage 3+, liver stage 3+, or skin stage 3 and lower GI or liver stage 2+ GVHD [2].

Histopathological examination of skin biopsies remains the primary method to confirm acute GVHD. Skin biopsy should be considered when eruption develops after HSCT even before engraftment, especially when other organ involvement is minimal. If the first skin biopsy is inconclusive, follow-up biopsy within a short time is helpful in the diagnosis of hyperacute GVHD [2].

In practice, there has been continuous search for a new diagnostic tool, involving immediate, objective, and noninvasive methods for the recognition of skin lesions in the course of acute GVHD, as well as dermatological changes in the skin that occur prior to the clinical manifestations of the disease.

Considering the high frequency of graft versus host disease in the hematologic management, dermoscopy can be regarded as a new routine diagnostic method. Dermoscopy (synonyms include terms such as* epiluminescence microscopy*,* skin surface microscopy*,* incident light microscopy*, and* dermatoscopy*) has, of late, made a name for itself as a greatly appreciated diagnostic method in dermatology [3]. Initially intended for the differential diagnosis of pigmented lesions, dermoscopy became more widespread in the 1990s. Dermoscopy has been used to the present day in assessing inflammatory dermatoses (inflammoscopy) and parasitic invasions (the so-called “entomodermoscopy”) and in cases of scalp disorders (trichoscopy), all in the follow-up to dermatological treatment [3].

The aim of the study was dermoscopic assessment of skin condition towards potential aGVHD involvement in patients after alloHSCT from starting the procedure until day 100.

## 2. Material and Methods

### 2.1. Patients

Thirteen consecutive patients aged >18 years, who received alloHSCT between September 2013 and June 2014, were included in the study. The transplant procedures were performed in the Department of Bone Marrow Transplantation and Oncohematology in Gliwice, Poland. The study protocol was approved by the Local Ethics Committee (KB/43041/13). All patients gave written informed consent. Details of patient and donor characteristics are shown in Table 1.

### 2.2. Transplantation Characteristics

Nine patients received conditioning based on irradiation (4–12 Gy) while the remaining 4 patients were treated with chemotherapy alone (Table 2). The conditioning regimen was myeloablative in 8 cases. All patients receiving transplantation from unrelated donors were treated with antithymocyte globulin. Peripheral blood was the source of stem cells in all patients.

### 2.3. Dermoscopic Procedure

Dermoscopy was performed by a certified dermatologist, an expert in dermoscopy, who is an integrated part of the transplantation team (Grazyna Kaminska-Winciorek) and trained on dermoscopy oncologist (Tomasz Czerw). All patients were examined clinically and dermoscopically on specific days before and after alloHSCT procedure [days 28 (±7), 56 (±7), and 100 (±7)], as well as each patient who developed cutaneous aGVHD diagnosed according to clinical criteria (Glucksberg scale) [1]. Moreover, during the occurrence of any skin lesions in all cases infections were excluded based on clinical symptoms, microbiology, viral tests, and laboratory assessment. Presence of CMV viremia in the serum was excluded in PCR method performed routinely in all patients every week after transplantation. Additionally all patients who received steam cells from unrelated donors were also monitored for EBV viremia every week. During skin lesion occurrence in all of them, CMV and EBV tests were negative.

Dermoscopic assessment of all monitored skin locations was performed using DermLite FOTO dermoscope (3Gen, LLC, Dana Point, California, USA) at 10-fold magnification. Minimal pressure was applied and ultrasound gel was used to preserve the morphology of blood vessels and ensure their better visualization. All dermoscopic images were captured and saved using a FotoFinder Medicam 800 HD Gold system (FotoFinder Systems GmbH, Bad Birnbach, Germany) in nonpolarized dermoscopy at 20-fold magnification. Skin locations most commonly affected in the course of acute GVHD were chosen for dermoscopy. In all patients, the selected areas of the skin were monitored dermoscopically, including the face (forehead, cheek, chin, and nose), décolleté, upper back, abdominal region, upper extremities including arms and forearms, hands (palmar and dorsal surfaces), and thighs in the above mentioned days of clinical and dermoscopic follow-up.

The selection of dermoscopic features included in the evaluation process was based on our preliminary observations and the data available in the literature [4–8].

The type of background and telangiectasias, presence of hyperpigmentation, and scaling were taken into account in the final dermoscopic description.

The following dermoscopic criteria were evaluated: (a) background: pale, pinkish, reddish, brownish, and yellow; (b) blood vessels: invisible (absent) or visible (present) as thin telangiectasias, thick telangiectasias, serpentine vessels, dotted vessels, and globular vessels; (c) scaling: absent or present; (d) hyperpigmentation: absent or present as reticular or homogeneous.

## 3. Results

### 3.1. The Incidence of Acute Graft versus Host Disease

Eight patients (62%) developed signs of acute GVHD. As assessed by clinical criteria, the severity of this complication was grade 1 (the majority of cases, *n* = 6), grade 2 (*n* = 1), or grade 3 (*n* = 1). In 6 cases, symptoms of skin involvement were present. Detailed characteristics are presented in Table 3.

#### 3.1.1. Results of Dermoscopy

**Figure 1:**
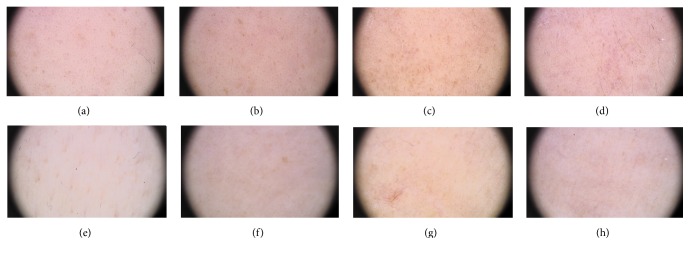
Case  7. In a 24-year-old female patient, 100-day dermoscopic follow-up has not revealed any skin reaction suggesting potential aGVHD characterized by pinkish or reddish background and increased number and visibility (presence) of blood vessels. These are examples of dermoscopic images taken on specific days of follow-up (−10, +32, +74, and +109) within the monitored locations (a, b, c, and d: forehead; e, f, g, and h: décolleté), which showed no dermoscopic features suggesting aGVHD. The picture is constantly homogeneous and remains stable in time.

In a subgroup of patients (6 patients: cases 1, 2, 3, 7, 8, and 13) the diagnosis of skin acute GVHD was not clinically confirmed and dermoscopic monitoring did not reveal any characteristic features of skin acute GVHD when all consecutive dermoscopic images were compared with each other (case 7, Figure 1).

**Figure 2:**
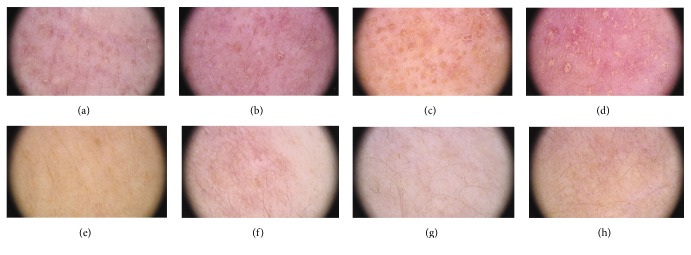
Case  4. A 20-year-old female patient developed aGVHD, stage 2° gut, on the 27th day after alloHSCT. Clinically no symptoms of skin aGVHD were found; however, dermoscopic follow-up revealed the following changes in the face compared to baseline images (a): marked reddish background with multiple telangiectasias (b), which periodically became less or more visible (c, d). Dermoscopic follow-up of the remaining skin locations, other than the face, showed no dermoscopic abnormalities suggesting an inflammatory skin condition in the course of potential aGVHD, for example, skin of décolleté on the specific days of monitoring [−15 (e), +49 (f), +74 (g), and +95 (h)].

These patients had no pinkish or reddish background and increased number of blood vessels or their telangiectatic dilation. In one patient, who was diagnosed with clinical gastrointestinal symptoms of acute GVHD, dermoscopy reverse-marked lesions in the face on day 49 with pinkish background and thin, multiple telangiectasias (case 4, Figure 2).

**Figure 3:**
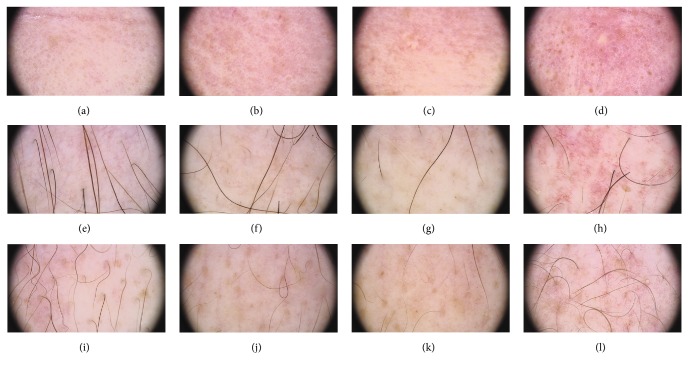
Case  10. A 42-year-old male patient developed skin aGVHD (stage 2°) within the back and arms on the 74th day. Dermoscopic images of the forehead (a, b, and c), décolleté (e, f, and g), and back (i, j, and k) obtained at the earlier time points of monitoring (−3, +32, and +53) revealed no telangiectasias. In all these locations, marked pinkish background with multiple telangiectasias was highly expressed in the late dermoscopic examination (+109) (d, h, and l), whereas clinical symptoms of aGVHD were clinically visible only on the back and arms. Dermoscopy showed pinkish background with features described as multiple teleangiectasias.

All 6 patients (cases 5, 6, 9, 10, 11, and 12) diagnosed with skin acute GVHD had clinical skin lesions parallel to the dermoscopic features of aGVHD. Dermoscopic findings suggesting skin acute GVHD in all 6 patients included pinkish or reddish background, well-visible, marked multiple thin telangiectasias (e.g., case 10, Figure 3, and case 12, supplemental figure in Supplementary Material available online at http://dx.doi.org/10.1155/2016/4535717).

Moreover, patients with clinically confirmed acute GVHD had additional dermoscopic features in selected regions, such as serpentine vessels, multiple thick telangiectasias, and multiple dotted vessels. Globular vessels were also seen in one patient (case 11).

In addition, all 13 patients were reported to have reticular hyperpigmentation and dotted vessels in the hands.

Scaling was reported only in one patient (case 5) with acute GVHD, especially in its late stages; another patient had perifollicular reddish, homogeneous dots (case 12).

All dermoscopic features in selected patients with acute GVHD were provided in supplemental table.

## 4. Discussion

Dermoscopy is one of well-known noninvasive methods allowing diagnosing not only melanocytic and nonmelanocytic lesions but general skin conditions as well [5, 6, 8, 9]. Nowadays, dermoscopy is compared to a stethoscope in the dermatologist's hands in diagnostic procedures used in general dermatology [5]. This diagnostic method improves the recognition of not only melanocytic and nonmelanocytic pigmented lesions but also inflammatory skin disorders [6, 7, 9, 10]. Dermoscopic assessment in transplant medicine has only been limited to casuistic cases including dermoscopic follow-up in a patient after near full facial transplant [4]. The first report, written by Kaminska-Winciorek et al. [4], implied that dermoscopy might be used as a noninvasive diagnostic and monitoring tool for acute graft rejection after facial transplantation [4]. Dermoscopic features suggesting potential acute facial rejection were described as the presence of telangiectasias with confluent, pinkish erythema. In the subsequent dermoscopic examinations, these lesions gradually resolved comparing with healthy own skin of the monitored recipient [4].

In most reports on dermoscopic assessment of general skin diseases, the morphology and architectural arrangement of blood vessels are clues to make the diagnosis, similarly to identification of nonmelanocytic tumors [11]. Lallas et al. [7] analyzed one hundred and fifteen dermoscopic images for the characteristic features of selected inflammatory diseases. Polygonal and linear vessels were typical for erythromelalgic rosacea [7], and multiple dotted vessels were found in seborrheic dermatitis [7]. Linear vessels can also be found in scaling spongiotic dermatitis/eczema accompanied by regular scales and in urticaria [12].

In our study, we observed linear vessels as well as multiple thin and thick telangiectasias in acute GVHD patients. In most of cases, they were located on reddish or pinkish background if they were inflamed and sometimes on a pale base. Dotted vessels in a regular arrangement over a light red background and white scales were also highly predictive of the diagnosis of psoriasis, whereas dermatitis more commonly showed yellow scales and dotted vessels in a patchy arrangement [9]. In our own study, dotted vessels were mainly seen in the region of the hands, most accentuated within skin markings in the inflammatory stage of acute GVHD.

Globular vessels were found only in one patient with aGVHD. Regularly distributed glomerular vessels, similar to those globular, have been described previously in advanced venous stasis dermatitis and insect bite reactions [12, 13]. Scaling as an additional dermoscopic feature has been observed sporadically and was associated with postinflammatory skin exfoliation, well reported in the literature [12].

Our results indicate dermoscopy as a potentially valuable tool for the diagnosis of aGVHD available for doctors of many specialities including not only dermatologists, but also hematologists and oncologists. Dermoscopic monitoring of the skin in patients after alloHSCT is easy to perform and safe and can be repeated at any point of the clinical follow-up without pain for the patient. On dermoscopy, highly suggestive features for aGVHD are multiple, telangiectatic vessels located within a pinkish or reddish background.

Till this time, there have been no reports on dermoscopic assessment of skin lesions after alloHSCT procedures. An attempt to correlate clinical abnormalities with dermoscopic images is innovative and it can be a source of valuable information for physicians performing dermoscopy as a noninvasive examination in order to differentiate skin lesions occurring in acute GVHD. This knowledge could improve the early recognition of aGVHD and minimize invasive procedures such as diagnostic biopsies.

The results of the study can contribute to the development of procedures that could be implemented in the management of patients suspected of skin acute GVHD in the nearest future.

## 5. Conclusions

This is only a pilot study and further research is still needed. Our results showed a correlation between the clinical picture and dermoscopic images in patients with aGVHD. We believe that dermoscopic monitoring should be systematically performed in all patients after alloHSCT to allow accurate identification of the early signs of acute GVHD.

## Supplementary Material

Supplemental table. Dermoscopic features of the skin in patients with skin acute GVHD in our study.
Supplemental figure. Case 12. A 41 year old male patient developed first clinical signs of skin aGVHD (stage 1°) on the 13th day after HSCT within the face and foot. Dermoscopic pictures of selected monitored locations showed marked pinkish and reddish background and blood vessels becoming wider, better visible and more numerous in the course of follow up (−5, +20, +34, +41, +62, +76, +104) (a–g: cheek; a1–g1: forehead; a2–g2: décolleté). Clinical follow up of the other monitored locations has not shown any clinical signs of aGVHD; however, dermoscopic examination revealed aggravated perifollicular reddish dots and marked telangiectasias in dermoscopic pictures obtained on the same days (a3–g3: back; a4–g4: abdominal region).

## Figures and Tables

**Table 1:** Patient and donor characteristics.

*Median patient age (range)*	42 (22–59) years
*Median donor age (range)*	38 (20–63) years
*Median time from diagnosis to transplantation*	0.84 (0.43–5.7) years
*Donor/patient sex, n (%)*	
Male/male	2 (15%)
Female/male	5 (38%)
Male/female	2 (15%)
Female/female	4 (31%)
*Donor/patient CMV status, n (%)*	
Negative/negative	0
Negative/positive	1 (8%)
Positive/negative	2 (15%)
Positive/positive	10 (77%)
*Donor, n (%)*	
HLA-identical sibling	9 (69%)
Matched unrelated	4 (31%)
*Diagnosis, n*	
AML	3
ALL	2
CML	1
SAA	1
OMF	1
MDS/MPS	1
Sézary syndrome	1
Lymphomas	2
CAEBV	1

**Table 2:** Transplantation procedure.

*Type of conditioning regimen, n (%)*	
Irradiation-based	8 (62%)
Chemotherapy-based	5 (38%)
Myeloablative	8 (62%)
Reduced intensity	5 (38%)
*Stem cell source, n (%)*	
Peripheral blood	13 (100%)
*Transplanted cells*	
Median CD34+ × 10^6^/kg	5.35 (4.1–12.58)
Median CD3+ × 10^7^/kg	22.9 (9.98–32.34)
*GVHD prophylaxis, n (%)*	
CsA/short course MTX	11 (84%)
CsA	1 (8%)
CsA/MMF	1 (8%)

**Table 3:** The incidence of acute GVHD.

Patient	Gender	Age	Donor (RD: related; URD: unrelated)	Grade of GVHD	Stage of GVHD: skin/liver/gut	Day of aGVHD onset
1	F	45	RD	0	/	/
2	M	52	RD	0	/	/
3	M	46	URD	0	/	/
4	F	20	RD	3	2° gut	27
5	F	24	URD	1	2° skin: face, décolleté	10
6	F	22	URD	1	1° skin: face	6
7	F	24	RD	0	/	/
8	F	52	RD	0	/	/
9	M	27	RD	1	1° skin: hands	24
10	M	42	RD	1	2° skin: back, arms	74
11	M	57	RD	1	2° skin: back, upper extremities	11
12	M	41	URD	1	1° skin: face, foot	13
13	M	59	RD	2	1° gut (died on the 77th day)	33

## Additional Points

Despite the different aetiopathogenesis, differential diagnosis of GVHD includes dermatitis, photodermatitis, and drug eruptions. There is no way to exclude the possibility of a transplant patient to develop one of the latter entities. Therefore it should be highlighted that the limitation of our study is the lack of control group of patients suffering from listed above entities. In the aspect of the lack of pathognomonic dermoscopic features, our dermoscopic findings cannot be used for differential diagnosis purposes, but only maybe as a follow-up tool.
