# Supplementary material for: Dermoscopic Follow-Up of the Skin towards Acute Graft-versus-Host-Disease in Patients after Allogeneic Hematopoietic Stem Cell Transplantation
Source: Biomed Res Int. 2016 Jun 30;2016:4535717. doi: 10.1155/2016/4535717 (PMC4944033; doi:10.1155/2016/4535717)
Supplement: Supplementary file 1 — Supplemental table. Dermoscopic features of the skin in patients with skin acute GVHD in our study. Supplemental figure. Case 12. A 41 year old male patient developed first clinical signs of skin aGVHD (stage 1°) on the 13th day after HSCT within the face and foot. Dermoscopic pictures of selected monitored locations showed marked pinkish and reddish background and blood vessels becoming wider, better visible and more numerous in the course of follow up (−5, +20, +34, +41, +62, +76, +104) (a–g: cheek; a1–g1: forehead; a2–g2: décolleté). Clinical follow up of the other monitored locations has not shown any clinical signs of aGVHD; however, dermoscopic examination revealed aggravated perifollicular reddish dots and marked telangiectasias in dermoscopic pictures obtained on the same days (a3–g3: back; a4–g4: abdominal region). [file 4535717.f1.pdf]

Supplemental table. Dermoscopic features of the skin in patients with skin acute GVHD.

Patients with aGVHD	Time of dermoscopic follow-up	Dermoscopic features in selected locations	Face	Décolleté	Back	Abdomen	Arms	Forearms	Dorsal hands	Hands	Thighs
Patient 5	Before alloHSCT	**background**	pale	pale	pale	pale	pale	pale	pale	pale	pale
		**vessels**	several thin teleangiectas	several thin teleangiectas	absent	absent	multiple thin teleangiectasias	absent	several thin teleangiectasias, multiple dotted vessels	multiple dotted vessels	multiple thin teleangiectasias
		**scaling**	absent	absent	absent	absent	absent	absent	absent	absent	absent
		**hyperpigmentation**	homogenous	absent	homogenous	absent	absent	absent	absent	absent	reticular
	After alloHSCT	**background**	pinkish	pinkish	brownish	brownish	pinkish	brownish	brownish	pale	brownish
		**vessels**	multiple thick to thin teleangiectasias	multiple thick to thin teleangiectas	several thin teleangiectasias	absent	several to multiple thick teleangiectas	several to multiple thin teleangiectasias	multiple thin teleangiectasias, several dotted vessels	several to multiple dotted vessels	multiple thin teleangiectasias
		**scaling**	absent	absent	present	present slight	absent	absent	present	absent	absent
		**hyperpigmentation**	homogenous	reticular	reticular	reticular, homogenous marked in skin markings	reticular	reticular	reticular , marked in skin markings	reticular, marked in skin markings	reticular, homogenous
Patient 6	Before alloHSCT	**background**	pale	pale	pale	pale	brownish	brownish	pale	pale	pale
		**vessels**	several thin teleangiectasias	several thin teleangiectasias	absent	absent	several thin teleangiectasias	absent	several thin teleangiectasias	multiple thin teleangiectasias, dotted vessels	several thin teleangiectasias
		**scaling**	absent	absent	absent	absent	absent	absent	absent	absent	absent
		**hyperpigmentation**	absent	reticular	absent	absent	reticular	absent	reticular	absent	reticular
	After alloHSCT	**background**	reddish	pinkish	pale	pale	pinkish	pale	pinkish	pale	brownish
		**vessels**	multiple thick to thin teleangiectas and serpentine vessels	multiple thick to thin, teleangiectasias and serpentine vessels	several thin teleangiectasias	several thin teleangiectasias	several to multiple thin teleangiectasias	absent	multiple thin teleangiectasias and serpentine vessels	multiple thin vessels and dotted vessels	several , thin teleangiectasias
		**scaling**	absent	absent	absent	absent	absent	absent	absent	absent	absent
		**hyperpigmentation**	absent	reticular	reticular	reticular	reticular	absent	reticular	absent	reticular
Patient 9	Before alloHSCT	**background**	pinkish	pale	pale	pale	pale	pale	pale	pale	pale
		**vessels**	several thin teleangiectasias	several thin teleangiectasias	several thin teleangiectasias	several thin teleangiectasias	several thin teleangiectasias	absent	absent	several dotted vessels	several thin teleangiectasias
		**scaling**	absent	absent	absent	absent	absent	absent	absent	absent	absent
		**hyperpigmentation**	absent	absent	absent	absent	absent	absent	absent	absent	absent
	After alloHSCT	**background**	pale	pinkish, brownish	brownish	brownish	yellow	brownish	pale	pale	brownish
		**vessels**	several thin teleangiectasias	multiple thin and thick, teleangiectasias	several thin teleangiectasias to several, slight visible teleangiectasias	multiple thin teleangiectasias , dotted vessels	several thin teleangiectasias	multiple to several thin teleangiectasias	multiple dotted vessels, multiple teleangiectasias	multiple to several dotted vessels	multiple thin teleangiectasias
		**scaling**	absent	absent	absent	absent	absent	absent	absent	absent	absent
		**hyperpigmentation**	reticular	reticular	reticular	absent	absent	absent	absent	absent	absent
Patient 10	Before alloHSCT	**background**	pinkish	pale	pale	pale	pale	pale	pale	pale	pale
		**vessels**	multiple thick teleangiectasias	multiple thin, teleangiectasias	several thin teleangiectasias	absent	several thin teleangiectasias	absent	several dotted vessels	absent	multiple thin teleangiectasias, several dotted vessels
		**scaling**	absent	absent	absent	absent	absent	absent	absent	absent	absent
		**hyperpigmentation**	absent	absent	reticular	absent	reticular	reticular	absent	absent	absent
	After alloHSCT	**background**	pinkish	pale	pale	pale	pinkish	pinkish	brownish	pinkish	pinkish

		**vessels**	multiple thin teleangiectasias , multiple thick teleangiectasias	several thin teleangiectasias	several thin teleangiectasias , multiple dotted vessels	absent	several thin teleangiectasias	several to multiple thin teleangiectasias	multiple thin, multiple dotted vessels	several thin vessels, several dotted vessels	multiple , thin teleangiectasias, multiple dotted vessels
		**scaling**	absent	absent	absent	absent	absent	absent	absent	absent	absent
		**hyperpigmentation**	reticular	reticular	homogenous localized within hair follicule, reticular	absent	reticular	reticular	reticular	absent	absent
Patient 11	Before alloHSCT	**background**	pinkish	pinkish	pinkish	pinkish	pale	pinkish, reddish	pinkish	pinkish, reddish	pinkish, reddish
		**vessels**	multiple thick teleangiectasia	multiple thick teleangiectasias	multiple thick teleangiectasias	multiple thick teleangiectasias	several thin teleangiectasias, several serpentine vessels, several dotted vessels	multiple thick teleangiectasias , globular vessels, serpentine vessels	multiple globular vessels	multiple thick teleangiectasias , globular vessels	multiple thick teleangiectasias , globular vessels, serpentine vessels
		**scaling**	absent	absent	absent	absent	absent	absent	absent	absent	absent
		**hyperpigmentation**	reticular	reticular	homogenous	homogenous	homogenous	homogenous	absent	homogenous	homogenous
	After alloHSCT	**background**	pinkish, reddish	pinkish	pale	reddish	pinkish, brownish, yellow	pinkish	pinkish	pinkish, reddish, brownish	pinkish, reddish
		**vessels**	multiple thick teleangiectasias	several to multiple thin to thick teleangiectasias	several thin serpentine vessels	thin several teleangiectasias	several thin teleangiectasias, several and multiple serpentine vessels, multiple dotted vessels	several thin teleangiectasias, serpentine vessels	multiple dotted vessels	multiple thin teleangiectasias, multiple dotted vessels	several thin teleangiectasias, several to multiple serpentine vessels, several to multiple dotted vessels
		**scaling**	absent	absent	absent	absent	absent	absent	absent	absent	absent
		**hyperpigmentation**	reticular	homogenous	homogenous	homogenous	homogenous	homogenous	absent	absent	homogenous
Patient 12	Before alloHSCT	**background**	pinkish	pale	pale	pale	pale	pale	pale	pale	pale
		**vessels**	multiple thin teleangiectasias	several thin teleangiectasias	several thin teleangiectasias	several dotted vessels	absent	absent	absent	absent	absent
		**scaling**	absent	absent	absent	absent	absent	absent	absent	absent	absent
		**hyperpigmentation**	reticular	absent	reticular	absent	reticular	reticular	absent	absent	absent
	After alloHSCT	**background**	pinkish, reddish, brownish	pinkish, reddish, brownish	pinkish, reddish brownish	reddish	pinkish , brownish	pinkish , brownish	pinkish reddish, brownish	pinkish	pinkish, brownish
		**vessels**	several to multiple thin teleangiectasias	several to multiple thin teleangiectasias , multiple thick teleangiectasias	several thin teleangiectasias, multiple thick teleangiectasias	several thin teleangiectasias, several dotted vessels	thin several to multiple teleangiectasias	several to multiple teleangiectasias	several to multiple thin teleangiectasias, multiple dotted vessels	several to multiple dotted vessels	multiple thin teleangiectasias, multiple dotted vessels
		**scaling**	absent	absent	absent	absent	absent	absent	absent	absent	absent
		**hyperpigmentation**	reticular	reticular	reticular	reticular	reticular mainly within hair follicles and skin markings	reticular mainly within hair follicles	reticular	homogenous mainly in skin markings	reticular

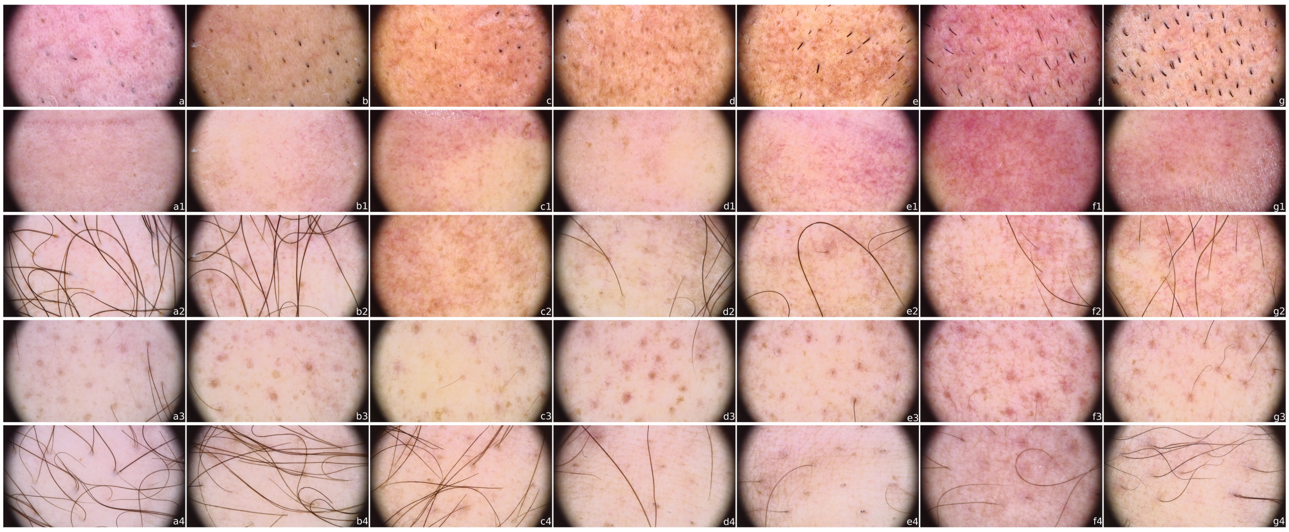

Supplemental figure. Case 12. A 41 year old male patient developed first clinical signs of skin aGVHD (stage 1º) on the 13th day after HSCT within the face and foot. Dermoscopic pictures of selected monitored locations showed marked pinkish and reddish background and blood vessels becoming wider, better visible and more numerous in the course of follow up (−5, +20, +34, +41, +62, +76, +104) (a–g: cheek; a1–g1: forehead; a2–g2: décolleté). Clinical follow up of the other monitored locations has not shown any clinical signs of aGVHD; however, dermoscopic examination revealed aggravated perifollicular reddish dots and marked telangiectasias in dermoscopic pictures obtained on the same days (a3–g3: back; a4–g4: abdominal region).
